# Application of Radiomics Model of CT Images in the Identification of Ureteral Calculus and Phlebolith

**DOI:** 10.1155/2022/5478908

**Published:** 2022-11-14

**Authors:** Qiuyue Yu, Jiaqi Liu, Huashan Lin, Pinggui Lei, Bing Fan

**Affiliations:** ^1^Department of Radiology, Jiangxi Provincial People's Hospital, The First Affiliated Hospital of Nanchang Medical College, Nanchang 330006, China; ^2^Department of Pharmaceutical Diagnosis, GE Healthcare, Changsha 410005, China; ^3^Department of Radiology, The Affiliated Hospital of Guizhou Medical University, Guiyang 550000, China

## Abstract

**Objective:**

To investigate the clinical application of the three-dimensional (3D) radiomics model of the CT image in the diagnosis and identification of ureteral calculus and phlebolith.

**Method:**

Sixty-one cases of ureteral calculus and 61 cases of phlebolith were retrospectively investigated. The enrolled patients were randomly categorized into the training set (*n* = 86) and the testing set (*n* = 36) with a ratio of 7 : 3. The plain CT scan images of all samples were manually segmented by the ITK-SNAP software, followed by radiomics analysis through the Analysis Kit software. A total of 1316 texture features were extracted. Then, the maximum correlation minimum redundancy criterion and the least absolute shrinkage and selection operator algorithm were used for texture feature selection. The feature subset with the most predictability was selected to establish the 3D radiomics model. The performance of the model was evaluated by the receiver operating characteristic (ROC) curve, and the area under the ROC curve (AUC) was also calculated. Additionally, the decision curve was used to evaluate the clinical application of the model.

**Results:**

The 10 selected radiomics features were significantly related to the identification and diagnosis of ureteral calculus and phlebolith. The radiomics model showed good identification efficiency for ureteral calculus and phlebolith in the training set (AUC = 0.98; 95%CI: 0.96–1.00) and testing set (AUC = 0.98; 95%CI: 0.95–1.00). The decision curve thus demonstrated the clinical application of the radiomics model.

**Conclusions:**

The 3D radiomics model based on plain CT scan images indicated good performance in the identification and prediction of ureteral calculus and phlebolith and was expected to provide an effective detection method for clinical diagnosis.

## 1. Introduction

Ureteral calculus is one of the most common diseases in the urinary system and is also a common disease-causing acute abdomen clinically, with a high incidence and recurrence rate, which adversely affects human health and life [1–5]. Patients with ureteral calculus need timely intervention and treatment. Therefore, an accurate diagnosis of ureteral calculus is of great importance [1]. Currently, various methods have been developed for the clinical detection of urinary calculus. The abdominal plain film shows a certain diagnostic effect on positive calculus, but it can barely detect negative calculus. Ultrasound can show calculus and urinary tract obstruction, but it is easily affected by the experience and manipulation of the doctors. However, computed tomography (CT) can clearly show the location, size, and quantity of calculus, urinary tract obstruction, and the changes in surrounding structures [1]. However, for some patients with a urinary tract infection or emaciation, the fat space surrounding the ureter disappears on plain CT scan images, and it may be difficult for radiologists to accurately track the path of the ureter [6]. In addition, ureteral calculus and phlebolith are not specific in CT images, showing high-density nodular shadows. Thus, it is difficult to distinguish them only by the image features observed by the naked eye [7].

As a novel and noninvasive technology, radiomics can mine the texture features in medical images in high-throughput and quantify the visual image data through a variety of advanced image processing methods, followed by objective and quantitative analysis [8]. Texture features include first-order features, shape and higher-order features (such as the gray-level cooccurrence matrix (GLCM), gray-levelrun-length matrix (GLRLM), gray-level size zone matrix (GLSZM), neighborhood gray-tone difference matrix (NGTDM), etc.). First-order features are analyzed based on the single pixel or voxel, which describe the texture of ROI by measuring the distribution of intensity levels of voxels and do not show the spatial relationship of voxels. Shape is a description of the geometric characteristics of ROI. Gray-level cooccurrence matrix (GLCM) belongs to the second-order gray histogram; it describes the spatial relationships of pairs of pixels or voxels with predefined grayscale intensities and distances in different directions. Gray-levelrun-length matrix (GLRLM) describes the spatial distribution information of continuous pixels with the same gray level in one or more directions. Gray-level size zone matrix (GLSZM), whose principle is similar to GLRLM, emphasizes that the counts of the number of zones of adjoining pixels or voxels with the same gray level form the basis of the matrix. A wider and flatter matrix means a more homogeneous texture. Different from GLCM calculation, GLSZM may be computed by the distance of different pixels or voxels that define the neighborhood. Neighborhood gray-tone difference matrix (NGTDM) describes the sum of differences between the gray level of a pixel or voxel and the average gray level of its neighboring pixels or voxels within a predefined distance [9, 10].

Currently, the radiomics model is being widely applied for medical imaging and is included in studying the images obtained by various examination equipment and various diseases, including those of tumors, such as malignancy evaluation, histological classification and grading [8, 11–15], wettability prediction, efficacy evaluation, and prognosis prediction [16–18]. Additionally, radiomics has shown promising results and a clinical application potential in predicting the prognosis of COVID-19 pneumonia patients [19], calculus component analysis, and evaluation of treatment effects [1, 3, 4, 20, 21]. In this study, we analyzed the features of CT image texture parameters between ureteral calculus and phlebolith through radiomics. We aimed to mine more quantitative information in CT images conducive to clinical diagnosis and identify an effective, convenient, and noninvasive detection method for clinical diagnosis.

## 2. Materials and Methods

### 2.1. Clinical Data

This study was approved by the ethics committee of Jiangxi Provincial People's Hospital, and all patients signed the informed consent form. Data of 122 patients with ureteral calculus and phlebolith who underwent an abdominal plain CT scan in Jiangxi Provincial People's Hospital were collected retrospectively analyzed to clarify the three-dimensional (3D) structure of the focus. Among the 122 cases, 61 had ureteral calculus, and 61 had phlebolith cases. Patients in the ureteral calculus group were 50.52 ± 15.02 years old (range = 19–84 years old), with a male/female ratio of 39 : 22. Patients in the phlebolith group were 54.15 ± 15.10 years old (range = 19–84 years old), with a male/female ratio of 34 : 27. All patients received abdominal plain CT scan, had complete image data with good quality, and were diagnosed through imaging and clinical diagnosis and treatment.

### 2.2. Examination Method and Scanning Parameters

All subjects in this study received routine abdominal plain CT scans. During the examination, patients were in a supine position and raised their hands above the head. Before scanning, patients were trained to reduce the interference caused by respiratory motion artifacts. Scanning parameters of the dual-source CT (Siemens, Germany): tube voltage = 120 kV, tube current = 150 mAs, scanning layer thickness and layer spacing = 5 mm, and reconstruction layer thickness = 1 mm (1.25 mm).

### 2.3. Image Segmentation

CT thin-section images can more clearly show the appearance of the lesions and contain more abundant texture information. Hence, axial CT thin-section reconstructed image was used for image processing and texture analysis in this study. All patient images were imported into the ITK-SNAP (Version 3.6.0, http://www.itksnap.org/pmwiki/pmwiki.php) software in DICOM format. Two abdominal radiologists (Doctor A has worked for five years and Doctor B has worked for more than 10 years) manually segmented the entire focus. First, the two doctors analyzed images from 25 randomly selected samples to assess repeatability between groups. Doctor A then repeated the same procedure. ICC values greater than 0.75 indicated good consistency of feature extraction, and the rest of the image segmentation was performed by doctor A. On axial CT images, ROI should be sketched along the edge of the focus and avoided for structures outside the focus. Finally, they sketched the 3D region of interest (ROI) layer-by-layer and in the whole range.

### 2.4. Extraction of Texture Features

Meanwhile, original images and segmented ROI files were imported into the Analysis Kit (AK; Artificial Intelligence Kit V3.0.2., Workbench2014, GE Healthcare). A total of 1316 texture feature parameters were extracted, including firstorder features (First_order), morphology (shape), GLCM, GLRLM, GLSZM, GLDM, NGTDM, and converted features based on logarithmic transformation (LOG; parameter Sigma selection 2.0, 3.0), wavelet transformation (Wavelet; Level 1), and local binary mode (LBP; Level 2, Radius 1.0, Subdivision select 1). Normalization processing of each feature value was performed based on *Z*-scores ((*x* − *μ*)/*σ*) to eliminate the deviation on the extracted feature value.

### 2.5. Feature Selection and Model Establishment

First, the Spearman rank correlation test was conducted to evaluate the correlation between each feature parameter. The feature parameters with a correlation greater than 0.8 were retained. Then, feature texture screening was performed according to the maximum correlation minimum redundancy (mRMR) criterion and the least absolute shrinkage and selection operator (LASSO) algorithm. The feature subset with the most predictability was selected to establish a 3D radiomics model. The ROC curve was used to evaluate the performance of the model. Finally, the clinical application of the model was evaluated by the decision curve.

### 2.6. Data Analysis

Statistical analysis was conducted by *R* software (Version: 3.4.4) and SPSS 22.0. General clinical measurement data were expressed as the mean ± standard deviation (*x* ± *s*). The “GLMMET” software package in R software was used for LASSO regression analysis. First, the optimized feature subset was identified by mRMR and the LASSO algorithm for model establishment. Then, the LASSO coefficients of the selected features were weighted, and the radiomics score (Rad-score) of each case was calculated. The prediction performance of the model was quantitatively evaluated by the AUC, accuracy, specificity, and sensitivity according to ROC curves. The higher AUC value indicated that the model had better prediction performance.

## 3. Results

**Figure 1:**
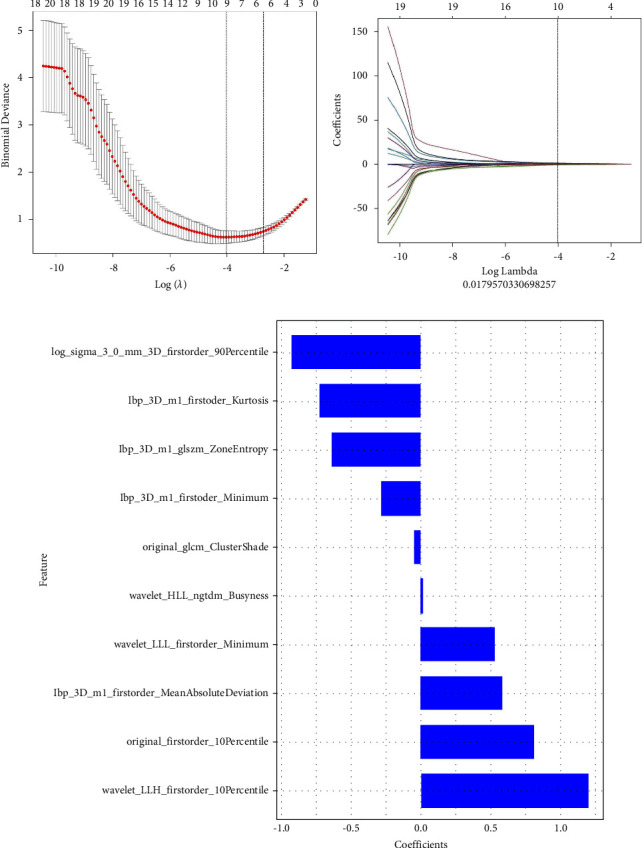
Radiomics features identified by the LASSO regression method. (a) The binomial deviation curve of the omics model with the parameter *λ*. The vertical axis represented the binomial deviation, and the horizontal axis represented log (*λ*) values; the parameter *λ* was adjusted to identify the optimized feature subset. The left vertical dotted line indicated log (*λ*) values corresponding to the optimized *λ*, and the number represented the number of selected features. (b) The changes in the radiomics model with *λ*. (c) Radiomics features screened based on the CT model and the corresponding coefficients.

CT images of 122 cases (61 cases of ureteral calculus and 61 cases of phlebolith) were included in this study. Patients were divided into the training group (86 cases) and the experimental group (36 cases) at the approximate ratio of 7 : 3. After mRMR and LASSO regression dimensionality reduction, redundant and irrelevant features were eliminated, and 10 feature parameters with the optimized predictability were identified, including seven firstorder statistical features, one GLCM feature, one GLSZM feature, and one NGTDM feature (Figure 1). The selected optimized feature subset was used to establish a radiomics model for diagnosis and prediction of ureteral calculus and phlebolith.

Rad-score was calculated according to the coefficient weighting of the selected features:

Rad-score = −0.635 ∗ lbp_3D_m1_glszm_ZoneEntropy + 0.809 ∗ original_firstorder_10Percentile + 0.586 ∗ lbp_3D_m1_firstorder_MeanAbsoluteDeviation + 1.201 ∗ wavelet_LLH_firstorder_10Percentile + −0.924 ∗ log_sigma_3_0_mm_3D_firstorder_90Percentile + −0.048 ∗ original_glcm_ClusterShade + −0.285 ∗ lbp_3D_m1_firstorder_Minimum + −0.725 ∗ lbp_3D_m1_firstorder_Kurtosis + 0.528 ∗ wavelet_LLL_firstorder_Minimum + 0.011 ∗ wavelet_HLL_ngtdm_Busyness + 0.075.

**Figure 2:**
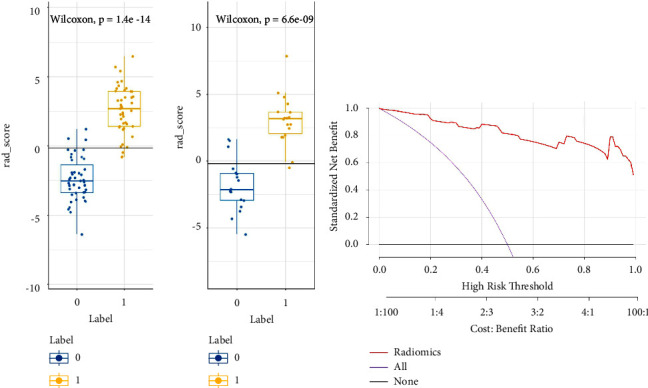
(a) Rad-score of ureteral calculus and phlebolith in the training group and the experimental group, and labels 0 and 1 corresponded to phlebolith and ureteral calculus, respectively. (b) The decision curve of the radiomics model.

**Figure 3:**
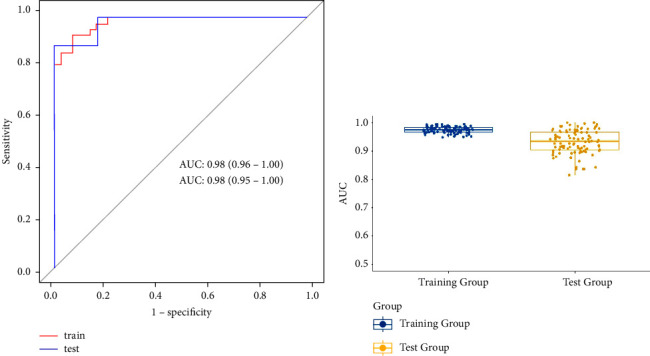
(a) The prediction model based on plain CT scan images. The AUC of the training group (*n* = 86) was 0.98, and the AUC of the experimental group (*n* = 36) was 0.98 (AUC: the area under the curve). (b) The AUC distribution of the training group and the experimental group in LGOCV.

In this study, the Rad-score of the training set and testing set showed a statistically significant difference (Figure 2), demonstrating that radiomics features were closely associated with the identification and diagnosis of ureteral calculus and phlebolith. Meanwhile, the Hosmer––Lemeshow test showed no statistically significant difference between the training set and testing set (*p* > 0.05), indicating good goodness of fit of the proposed radiomics model. The accuracy, sensitivity, specificity, and AUC in the training group and the experimental group were 93.02%,88.89%, 93.02%, and 94.44% and 93.02%, 83.33%, 0.98, and 0.98, respectively. Additionally, the reliability of our results was verified through the leave-group-out cross-validation (LGOCV) test. The average AUC of the training group and the experimental group was 0.975 and 0.932, respectively. Therefore, the results showed a good diagnosis and prediction performance of the radiomics model in the training group and the experimental group (Figure 3; Table 1).

## 4. Discussion

Ureteral calculus, a common cause of acute renal colic, can cause abdominal pain, hematuria, urinary tract obstruction, urinary tract infection, and damage to renal function over a long time and thus adversely affect human health [1, 22]. Phleboliths do not cause any discomfort and do not need any treatment. Therefore, immediate diagnosis and treatment of ureteral calculus are essential [23]. Currently, CT is regarded as the “gold standard” for the diagnosis of ureteral calculus because of its simple, rapid, and non-invasive operation and multiplanar thin-layer reconstruction [7, 24]. However, conventional diagnosis mainly depends on the relationship between high-density focus and ureter. The high-density focus in the ureter is diagnosed as ureteral calculus. But, when the fat contrast between the ureter and the surrounding structures (e.g., blood vessels, accessories, intestinal tubes) is absent, it may be difficult to assess the relationship between the high-density focus and the ureter on unenhanced CT images [6, 7, 25]. In this case, patients usually need additional examinations (e.g., retrograde urography, enhanced CT, CTU, or ureteroscopy) to establish the diagnosis, which increases the additional cost and potential risk. Additionally, accurate diagnosis may also be influenced by the subjectivity and clinical experience of the doctors.

Texture features can be employed to capture the appearance features of ROI in the images and analyze the distribution heterogeneity of elements in ROI. Meanwhile, texture features, without additional costs and examination, can objectively and quantitatively reflect the information in the images that cannot be identified by the naked eye and have been widely applied in medicine [9, 24]. AK mainly improves the accuracy of texture classification through the texture features statistics method. In this study, using AK software, 1316 texture feature parameters were extracted from 122 objects. After dimension reduction, 10 texture features with predictive significance were identified by the LASSO method to establish a 3D radiomics prediction model, including first-order and high-order features.

The first-order feature describes the texture by measuring the distribution of voxel intensity in the ROI and does not show the spatial relationship of voxels in the images. This study found that seven feature parameters in the model were first-order features (e.g., 10 percentile, mean absolute deviation, 90 percentile, minimum, kurtosis), with the corresponding coefficient of wavelet_LLH_first order_10 Percentile being the largest. The results demonstrated that the gray intensity distribution of ureteral calculus and pelvic phlebolith was different, suggesting that first-order features were of great significance for the diagnosis of ureteral calculus. Additionally, significant differences were observed in some high-order features, including original_glcm_ClusterShade, lbp_3D_m1_glszm_ZoneEntropy and wavelet_HLL_ngtdm_Busyness. GLCM describes the spatial relationship of pixels or voxel pairs with certain gray intensities and predefined distances in different directions. Cluster shade represents the uniformity and skewness of voxels in an image. GLSZM reflects the uniformity of image texture. The randomness of the distribution of the internal coefficients in grayscale images at the intensity level is evaluated by regional entropy. Busyness describes the high spatial frequency of pixel or voxel intensity changes. Therefore, the higher busyness indicates higher spatial frequency of ROI intensity changes in the lesions. The ROI is composed of many small areas with significantly different gray levels. These differences in high-order features indicated the heterogeneity of the spatial structure of the ureteral calculus [8, 9, 21, 26, 27].

Ureteral calculus has a concentric, layered, and microcrystalline structure [28, 29], suggesting that the microstructure of calculus is not single [30]. Meanwhile, phlebolith adheres to the venous wall and is formed by laminar fibrosis, central necrosis, calcium deposition, and calcification of mural thrombus [23, 31], and ureteral calculus is related to the long-term supersaturation of a compound in urine [30, 32, 33], suggesting that the mechanism of phlebolith and ureteral calculus is different. Traubici et al. [34] found that phlebolith and ureteral calculus were different on X-ray plain films. Phlebolith had radioactive light transmission centers, which were not found in CT images. Meanwhile, macroscopically visible lesion morphology, marginal sign, and comet tail sign on unenhanced CT can distinguish ureteral calculus from phlebolith [7, 23, 29, 35, 36]. Tanidir et al. [37] measured the density and volume of ureteral calculus and phlebolith on CT images and found differences in CT images when the volume was 171 mm^3^ and the density was 643 HU (sensitivity = 75% and 75%, specificity = 100% and 93%). Additionally, Lee et al. [25] applied the artificial neural networks (ANN) method to analyze the morphological features of the ureteral calculus and phlebolith and found some differences. Meanwhile, they also analyzed the internal texture features and found that skewness and difference histogram variation could distinguish ureteral calculus from phlebolith. De Perrot et al. [38] reported that radiomics and machine learning could accurately distinguish renal calculus and venous calculus using the low-dose CT (AUC was 0.902, PPV was 81.5%, and NPV was 90.0%). In a radiomics study by automatic segmentation, Homayounie [2] found that the combination of 3D_log_sigma of short-run low gray-level emphasis, exponential of run variance, run entropy (GLRLM features), and GLCM_maximal correlation coefficient could effectively diagnose the renal calculus (AUC = 0.84, 95% CI = 0.78–0.89). The combination of GLCM_inverse difference moment normalized, NGTDM_exponential of coarseness and GLRLM_3D_log_sigma of short-run low gray-level emphasis showed better diagnostic performance (AUC = 0.9 and 95% CI = 0.85–0.93). Furthermore, Mohammadinejad et al. 4 has reported that the semiautomated radiomic analysis of urinary stones is able to provide similar accuracy compared with manual measurements for predicting urinary stone passage. Therefore, studies in this line of research present the potential to, conservatively, improve the quality of life of patients with calculus. The findings in these studies are consistent with those in the present study, further suggesting a difference in the microstructure of ureteral calculus and phlebolith.

In this study, the texture features of plain CT images of ureteral calculus and phlebolith were compared and analyzed by radiomics and model establishment. The results showed significant differences in the feature parameters between ureteral calculus and phlebolith, indicating a good identification efficiency of the proposed radiomics model (the AUC of the training group and the experimental group was 0.98 and 0.98, the PPV was 93.0% and 85.0%, and the NPV was 93.0% and 93.8%, respectively). Meanwhile, the Hosmer––Lemeshow test and decision curve were performed to further verify the stability of the diagnostic performance of the proposed radiomics model. Therefore, the radiomics model is feasible to quantitatively distinguish ureteral calculus from pelvic phlebolith, showing the potential application of the radiomics model outside the field of cancer.

Previous studies have reported that the identification efficiency of radiomics based on different CT suppliers was stable, without significant differences [2, 10, 11, 39]. Hence, the data in this study were obtained from CT examination equipment of different manufacturers. The established model based on the optimized texture features extracted from these data showed good prediction performance. Moreover, the LGOCV test verified that the prediction performance of the proposed model was reliable, indicating that the proposed radiomics model had certain robustness. Meanwhile, the proposed model preliminarily showed the potential correlation between the feature parameters in each prediction subset and the prediction of ureteral calculus, which was not reported previously. However, the exact correlation between each feature parameter and ureteral calculus needs further study.

This study had some limitations: (1) it was a retrospective study with limited samples; there may be selection bias; (2) classification research was not performed on ureteral calculus, while the texture parameters of calculus in different categories are different; and (3) this is a single-center study, and multicenter research is expected in the future.

## 5. Conclusions

The radiomics model established in this study, based on plain CT images, showed good predictive performance for ureteral calculus and phlebolith, which was expected to provide an effective and convenient detection method for clinical diagnosis to alleviate the heavy clinical pressure.

## Figures and Tables

**Table 1:** The diagnostic performance of the radiomics model in the training group and the experimental group.

Group	AUC	Accuracy	Sensitivity	Specificity	Pos.pred.value	Neg.pred.value
Train	0.98 (95% CI:0.96–1.00)	0.930	0.930	0.930	0.930	0.930
Test	0.98 (95% CI:0.95–1.00)	0.889	0.944	0.833	0.850	0.938

AUC = area under the ROC curve; CI = confidence interval.

## Data Availability

The datasets used and analyzed during the current study are available from the corresponding author upon request.
